# SARS-CoV-2 Threat Perception and Willingness to Vaccinate: The Mediating Role of Conspiracy Beliefs

**DOI:** 10.3389/fpsyg.2021.672634

**Published:** 2021-08-19

**Authors:** Alexandra Maftei, Andrei Corneliu Holman

**Affiliations:** Faculty of Psychology and Education Sciences, Alexandru Ioan Cuza University, Iaşi, Romania

**Keywords:** COVID-19, vaccination hesitancy, threat perception, conspiracy beliefs, SARS-CoV-2, planned behavior

## Abstract

In the current exploratory study, we investigated the willingness of participants to vaccinate against the novel coronavirus [severe acute respiratory syndrome coronavirus 2 (SARS-CoV-2)] that has shaken up the world since the beginning of 2020. More specifically, we tested the mediating role of conspiracy beliefs (CBs) on the relationship between threat perception (TP) and willingness of participants to vaccinate against coronavirus disease 2019 (COVID-19), along with a series of associated demographic variables. Overall, 40% of our sample expressed total rejection of the COVID-19 vaccine. Our results suggested no significant differences in gender, age, educational level, and vaccine acceptance or hesitancy of participants. The results also indicated that CBs partially mediated the relationship between TP and willingness of participants to vaccinate. The current findings are discussed within the theory of planned behavior (TPB) framework and their importance for public health communication and practices and building public trust within the global fight against COVID-19. We considered the present results as a valuable starting point in understanding the psychological constructs related to the extended model of TPB and other personal factors and addressed the attitudinal roots that shape the acceptance and rejection of COVID-19 vaccination.

## Introduction

Understanding the motivational roots of vaccine hesitancy of severe acute respiratory syndrome coronavirus 2 (SARS-CoV-2), one of the most controversial current issues, is essential for public health communication, strategies, and practices in the global fight against coronavirus disease 2019 (COVID-19). As the WHO already stated in 2019 (World Health Organization, 2019), vaccine refusal is a worldwide health threat. Previous related studies suggested a generally low acceptance of the COVID-19 vaccine (Fisher et al., 2020; Neumann-Böhme et al., 2020; Paul et al., 2020; Taylor et al., 2020; Guidry et al., 2021). In Romania, for example, a national survey from January 2021 suggested that 30% of Romanians expressed their rejection toward the COVID-19 vaccine, 40% of them motivating their refusal due to the lack of trust in its efficiency (GSSC Avangarde, 2020). This percentage is lower than the data provided by IPSOS in October (IPSOS, 2020), which initially suggested that only 57% of Romanians expressed their willingness to vaccinate, with higher percentages among men (68%). Currently (i.e., February 2021), around 700,000 people got vaccinated in Romania, ranking among the highest vaccination rates in Europe (Bloomberg, 2020). The Romanian vaccination national campaign strategy involved public names and religious figures in a continuous attempt to increase vaccine acceptance.

Since the COVID-19 outbreak, scientists worldwide have engaged in an unprecedented race to develop an effective vaccine to solve this devastating global health issue that has already killed more than 2.2 million people. Among the most common predictors of the rejection of vaccination are beliefs in COVID-19 conspiracy theories (CTs), lower educational levels, poor knowledge of COVID-19, low income, younger age, and female gender (Al-Mohaithef and Padhi, 2020; Fisher et al., 2020; Paul et al., 2020; Taylor et al., 2020; Murphy et al., 2021). Factors generally associated with acceptance/approval of COVID-19 vaccine (and explicit the willingness of people to vaccinate) are higher perceived risk of COVID-19, older age, trust in scientific experts, and sufficient general information related to the COVID-19 vaccine (Sherman et al., 2020; Guidry et al., 2021; Thaker, 2021). The systematic review of Lin et al. (2020) on the currently available data suggests that the regional infection rates and the personal experience of COVID-19 were inconclusive in terms of impact on vaccine receptivity.

### Conspiracy Beliefs, TP, and COVID-19

The belief of people in CTs (i.e., the “attempts to explain the ultimate causes of significant social and political events and circumstances with claims of secret plots by two or more powerful actors;” Douglas et al., 2019, p. 4) related to COVID-19 were among the most commonly associated factors to negative attitudes toward the SARS-CoV-2 vaccine (Bertin et al., 2020; Earnshaw et al., 2020; Murphy et al., 2021; Sallam et al., 2021) and vaccination in general (Lewandowsky et al., 2013; Jolley and Douglas, 2014). Believing in COVID-19 CT generally undermines the engagement in public health recommendations and policies aimed to contain the spread of the novel coronavirus (Allington et al., 2020; Biddlestone et al., 2020; Bierwiaczonek et al., 2020; Earnshaw et al., 2020; Maftei and Holman, 2020; Romer and Jamieson, 2020; Teovanović et al., 2020). For example, Romer and Jamieson (2020) found that beliefs in COVID-19-related CTs were inversely related to the perceived COVID-19 threat and COVID-19 vaccination intentions. Thus, the higher the conspiracy belief (CB) levels, the lower the perceived threat of the pandemic. Similar conclusions were suggested by several other studies, such as the ones conducted by Imhoff and Lamberty (2020), Bertin et al. (2020), and Barua et al. (2020). They suggested that individuals high in COVID-19 CBs were more unlikely to adopt preventive measures and less likely to get vaccinated (Freeman et al., 2020).

Threat perception concerning SARS-CoV-2 was documented as a significantly associated factor of COVID-19 compliance to preventive measures (e.g., social distancing, hand washing, and wearing a mask) (Pan et al., 2020; Xu et al., 2020) and acceptance of COVID-19 vaccination (Fu et al., 2020). Previous findings suggest that one of the routes of this influence is through some of the psychological factors described by the Theory of Planned Behavior (TPB; Ajzen, 2011), which has been extensively used in predicting health-related behaviors. TPB states that attitude, subjective norm, and perceived behavioral control shape behavioral intentions of people: behavior is predicted by intention, and intentions are subject to attitudes of individuals, which in turn, are guided by behavioral beliefs (i.e., what individuals believe about the probable consequences of their behavior), normative beliefs (i.e., beliefs related to the normative expectations of other individuals), and control beliefs (i.e., what people believe about other factors that might impact the performance of their behavior) (Arafat et al., 2018). TP was explored in relation to the TPB components in a recent study by Adiyoso and Wilopo (2020). Their findings suggested that TP might predict behavioral intentions related to COVID-19 when mediated by attitudes and social norms, in line with previous findings that suggested the strong impact of risk perception in health-related contexts (Schmiege et al., 2009). The authors highlighted the need to consider behavioral promotions that increase risk perception in shaping public policies related to COVID-19 prevention.

In contrast, TP through perceived risk was also found to be positively correlated to CB in a recent study by Kim and Kim (2020). However, Alper et al. (2020) found no significant associations between COVID-19 CBs and compliance to preventive measures and a significant negative correlation between TP and adherence to preventive measures. Similar previous studies suggested that the infection TP seems to be a significant predictor of vaccination acceptance, in general (Nguyen et al., 2011; Gidengil et al., 2012).

Although results connecting COVID-19 TP, CB, and acceptance of vaccination might seem mixed, previous (pre-pandemic) studies generally suggest that CBs tend to decrease the perceived threat in such challenging times as the pandemic or suggest other means of confronting it, though the threats at individual and community levels might motivate action (Strecher and Rosenstock, 1997).

### The Present Study

In the present research, we explored the associated factors of vaccine acceptance within the extended model of TPB. Given the previous findings related to the significant associations related to beliefs in CT and COVID-19 TP, the focus of our research was on these variables, in addition to a series of demographical variables. Therefore, in the current exploratory study among Romanian adults, we examined the associations between willingness of participants to vaccinate against SARS-CoV-2 and gender, age, and educational level.

Previous findings suggested that beliefs in CT related to COVID-19 would generally lower the chances of accepting the vaccine. At the same time, higher COVID-19 TP levels seem to increase the willingness of people to vaccinate. Therefore, we tested a mediation model, assuming that COVID-19 CBs would mediate the relationship between TP and willingness to vaccinate of participants.

Although most studies generally considered TP and beliefs in CT as separate, parallel factors associated with COVID-19 vaccination intentions, our primary focus was on the idea that higher levels of COVID-19 TP might lower the chances to believe in CT. Previous studies focused on the Romanian population also support this idea, suggesting that the lower perceived threat of COVID-19 was associated with higher chances to hold CBs related to this pandemic (Maftei and Holman, 2020). One potential explanation might be related to the fact that those who feel most threatened by this novel virus might also process the COVID-19 information more rationally and more carefully; therefore, they might also be less influenced by COVID-19 conspiracies. For example, previous research in similar health-threatening situations (i.e., the 2009 N1H1 pandemic) suggested that people who considered the pandemic as a low threat (i.e., they perceived themselves as having a low risk of infection) were more likely to disobey public health strategies for managing the influenza pandemic and less likely to vaccinate (Taha et al., 2013).

Finally, our assumption that higher levels of COVID-19 TP might lower the chances to believe in CT might also be explained by the optimism bias (i.e., “the mistaken belief that our chances of experiencing negative events are lower than predicted or than those faced by our peers;” Pascual-Leone et al., 2021, p. 423). More specifically, individuals who do not feel threatened by the COVID-19 might generalize their feeling of security at the community level, thus being more prone to consider the restrictions aimed to contain the novel coronavirus as excessive and to suspect them to be motivated by hidden interests and part of larger conspiracies (Floyd et al., 2000; Pascual-Leone et al., 2021).

## Materials and Methods

### Participants

Our final cross-sectional sample was formed by 247 adults from the northeastern side of Romania, aged 18–70 years (*M* = 30.23, SD = 12.21), most of them being women (78%) (Table 1). Our sample covered two major cities from the northeastern side, i.e., Iaşi (the second largest city in Romania, with a population of around 375,000 people) and Bacău (with a population of around 145,000 people). The only inclusion criterion was related to age (>18 years). Four participants from our initial sample were excluded due to incomplete demographic information (i.e., gender and educational level).

**Table 1:** Demographic characteristics.

**Variables**	***N* (%)**
1. Age, mean (SD)	30.23 (12.21)
2. Gender	
Female	193 (78.1)
Male	54 (21.9)
3. Education level	
High school degree	133 (53.8)
University degree	114 (46.2)
4. Relationship status	
Single	60 (24.3)
Married	66 (26.7)
In a domestic partnership	121 (49)
5. Place of residence	
Urban	247 (100)
6. Region of residence	
North East of Romania	247 (100)
7. City of residence	
Iaşi	163 (66)
Bacau	84 (34)

### Research Procedure

We conducted a web-based survey in October 2020. At that moment, there were no approved COVID-19 vaccines, though several companies (e.g., BioNTech/Pfizer and Moderna) announced the release of efficient vaccines until the end of 2020. Therefore, the chances for a safe, efficient vaccine in the following 6 months (i.e., our dependent variable) were announced as high. The present cross-sectional survey used an online questionnaire distributed *via* social media platforms and communication groups (i.e., Facebook, WhatsApp, and LinkedIn). All participants voluntarily participated in this study and gave written informed consent following the 2013 Declaration of Helsinki. They were informed that the information they provided would remain anonymous and confidential and that they could retire from this study at any time. The time needed to answer all the research questions was around 15 min. The research protocol was designed following the ethical requirements specific to the faculty where the authors are affiliated.

### Research Instruments

The COVID-19 TP scale (Imhoff and Lamberty, 2020) consists of four items measured on a 7-point Likert scale, ranging from 1 (not at all) to 7 (very much). Example items include *To what extent are you currently worried about the spread of coronavirus?* and *To what extent do you currently feel threatened by the spread of coronavirus?* We calculated the total score for this variable by adding each value (i.e., the sum of scores), and Cronbach's alpha indicated good internal consistency (α = 0.860). The higher the score, the higher the COVID-19 TP reported by the participants.

The COVID-19 CT belief scale (Biddlestone et al., 2020) consists of 10 items measured on a 5-point Likert scale ranging from 1 (totally disagree) to 5 (totally agree). Example items include *coronavirus was purposefully created in, and released from, a biochemistry lab in Wuhan, China*; *Pharmaceutical companies created and released coronavirus in order to sell their medications and vaccines;* and *The new world order have finally found their most effective means of controlling populations through the release of coronavirus*. We calculated the total score for this variable by adding each value (i.e., the sum of scores), and Cronbach's alpha indicated good internal consistency (α = 0.912). The higher the score, the higher the COVID-19 CBs reported by the participants.

Finally, our dependent variable was formed by the answers given to the following question: “If a COVID-19 vaccine would be freely available in the next 6 months, would you get vaccinated?” Participants answered on a 5-point Likert scale ranging from 1 (definitely not) to 5 (definitely yes). The higher the score, the higher the willingness of participants to get vaccinated against COVID-19.

A demographic scale assessed the age, gender, marital status, and educational level of participants. All instruments were first pretested in a similar sample of adults (*N* = 32, *M* age = 29.06), and no difficulties were reported related to the items used within the questionnaires.

We used the SPSS 22.0 program (IBM® Corp. Released 2013, Armonk, NY) and the Hayes (2013) SPSS macro program PROCESS to to analyze our data.

## Results

Means, SD, and correlations between the main variables are presented in Table 2. Overall, 40% of our sample expressed total rejection for the COVID-19 vaccine (i.e., they answered “definitely not” to the question concerning vaccination intentions), while 21.9% of our sample expressed total acceptance/confirmed their vaccination intentions by choosing the “definitely yes” answer.

**Table 2:** Means, SD, and correlations between the main variables (*N* = 247).

	**Mean**	**Standard deviation**	**Min**	**Max**	**1**	**2**	**3**
1. Threat perception	12.10	4.50	3	21	–		
2. Conspiracy beliefs	21.56	9.44	9	45	−0.274^**^	–	
3. Vaccination intentions	2.51	1.61	1	5	0.266^**^	−0.438^**^	–
4. Age	30.23	12.21	18	70	0.223^**^	0.008	0.003

** *p < 0.001*.

We found significant correlations between vaccination intentions (VAX), TP, and CB. More specifically, higher TP levels and lower levels of CB were associated with higher levels of vaccination approval. Additionally, TP significantly and negatively correlated with CB, suggesting that individuals who expressed higher TP levels were less prone to engage in COVID-19 CBs. Also, we found a positive association between age and TP, suggesting that older participants expressed significantly higher levels of COVID-19 TP.

For a more comprehensive view of the potential differences related to gender and education levels of participants, we performed the independent *t*-test. The results of *t*-test indicated neither significant gender differences between intentions of participants related to COVID-19 vaccination (VAX), *t*_(245)_ = 0.670, *p* = 0.503, nor significant differences depending on their educational level, *t*_(245)_ = −0.707, *p* = 0.480.

**Figure 1:**
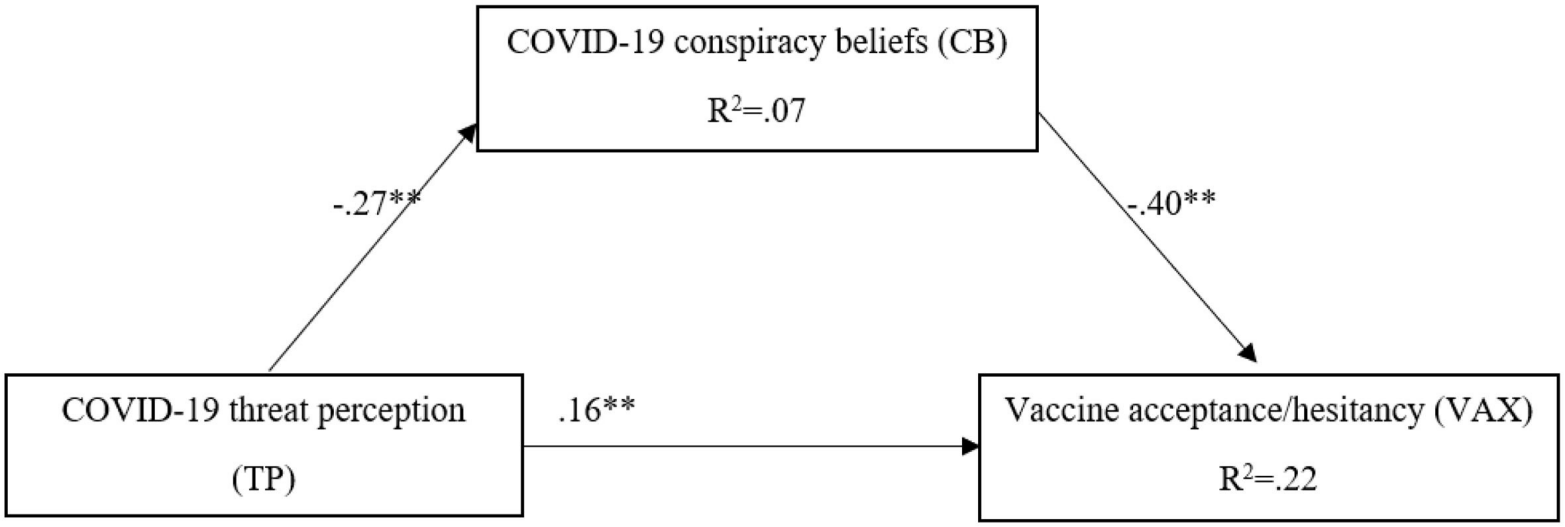
The mediating effect of CB on the relationship between TP and VAX. The values represent standardized coefficients; **p < 0.001; *p < 0.01.

The theoretical hypothesis model was tested by estimating the 95% CI for mediation effects with 5,000 bootstrap samples. In the present research, we used Model 4 to explore the mediating effect of beliefs of participants in CT related to COVID-19 on the relationship between TP and willingness of participants to vaccinate against this novel coronavirus (VAX). The total effect of TP on VAX (i.e., without taking into account the mediator) was significant as follows: *b* = 0.09, β = 0.27, *t*_(245)_ = 4.31, *p* < 0.001, SE = 0.02, 95% CI [0.05, 0.14], *R*^2^ = 0.08. Furthermore, the effect of TP on CB was also significant as follows: *b* = −0.05, *t*_(245)_ = −4.46, *p* < 0.001, SE = 0.13, 95% CI [−0.83, −0.32]. In the model that included both TP and CB as predictors of VAX, CB emerged as a significant predictor (*b* = −0.07, *t*_(245)_ = −6.70, *p* < 0.001, SE = 0.01, 95% CI [−0.08, −0.05]). Also, the direct effect of TP on VAX was still significant (*b* = 0.05, *p* = 0.008, SE = 0.02, 95% CI [0.01, 0.09]). However, the indirect effect of TP on VAX through CB was also significant (*b* = 0.03, bootstrapped 95% CI [0.02, 0.05]), suggesting that the relationship between TP and VAX is partially mediated by CB. Figure 1 shows the mediation model with the values of the standardized coefficients of each of the relationships between the variables.

In order to examine the statistical power of this study, we performed a sensitivity analysis G^*^Power 3.1 (Faul et al., 2007). Our aim was to identify the minimum size of the effect that our sample size can detect as significant in the type of research design that we used, focusing on the relationships between two independent variables (i.e., TP and CB) and a dependent variable (i.e., VAX). The size of the smallest effect between the variables in our model, which we examined through regression analyses, is Cohen's *f* ^2^ = 0.075. With an alpha = 0.05 and power = 0.95, the minimum effect size of such a relationship that would be detectable with our sample of 247 participants is *f* ^2^ = 0.05. All the effects in our model are above the value of the predicted minimal detectable effect size, which suggests that this study is appropriately powered.

## Discussion

Our exploratory findings suggested no significant effects of gender, age, and educational level on vaccine acceptance or hesitancy of participants. Therefore, our results contradict most of the previous-related findings that generally suggested that lower educational levels, younger age, and female gender (Al-Mohaithef and Padhi, 2020; Fisher et al., 2020; Paul et al., 2020; Taylor et al., 2020; Murphy et al., 2021) are usually associated with lower rates of acceptance of COVID-19 vaccination. However, our results are based on a low sample (i.e., the most significant limitation of this study), compared with the sample sizes of these studies.

Our results also suggested that CBs partially mediated the relationship between TP and willingness of participants to vaccinate. Previous research indicated that higher levels of perceived risk and lower levels of CB are generally associated with the explicit willingness of people to vaccinate against SARS-CoV-2 (Adiyoso and Wilopo, 2020; Bertin et al., 2020; Earnshaw et al., 2020; Sherman et al., 2020; Guidry et al., 2021; Murphy et al., 2021; Sallam et al., 2021; Thaker, 2021). Therefore, our findings confirm these associations and add to the scientific background related to understanding the hesitancy of COVID-19 vaccination.

We found that higher CBs are associated with lower risk perception related to the novel coronavirus. As research already suggested, people who usually consider SARS-CoV-2 a product of “Big Pharma,” or a very mild condition, such as the flu, or even more, they consider COVID-19 does not even exist, and governments invented it in a battle to control the world (Andersen et al., 2020) also perceive a low risk of infection, i.e., low TP. The findings in this study suggested that higher levels of perceived threat seem to have a direct effect on vaccination intention, as well as an indirect effect, through lowering the CBs of people. Therefore, our results highlight the necessity for health policies and governmental information strategies to actively advocate against the growing CB around SARS-CoV-2 and more importantly, about the vaccine. More importantly, given the significant association between TP and age, our findings highlight the need to address these targeted health and communication strategies mostly to younger people.

Orosz et al. (2016) proposed several effective strategies for reducing CBs within the primary TPB assumption. Among the strategies they suggested are those that imply using rational and ridiculing arguments targeting the link between the object of belief (COVID-19, in our case) and its characteristics (with a focus on its origins and symptoms). Moreover, concerning attitudinal variables, CBs seem positively related to perceived lack of control and lack of trust in other people and the authorities (Whitson and Galinsky, 2008; Brotherton et al., 2013), which we already pointed out as significant variables related to COVID-19 health-related behaviors. Extrapolating the solutions of Orosz et al. (2016) to the COVID-19 vaccination context, we might find it useful to point out the logical flaws of the links between CBs surrounding COVID-19 and vaccination procedures and potential adverse effects. According to Orosz et al. (2016), “the rational strategy is related to the central route of persuasion in which an individual holding CT (i.e., conspiracy theories) beliefs evaluates the pros and cons of the rational arguments and estimates the fit of these detailed arguments to the preexisting value system” (p. 2). Another strategy proposed by the authors is self-distancing, i.e., increasing the distance between conspiracy believers and other individuals who engage in similar practices, using specific arguments to increase the cognitive dissonance of an individual. However, these specific strategies need further exploration to properly consider them as potential effective strategies to increase positive COVID-19 health-related behaviors.

There are several ways that policymakers and governments, in general, might address vaccine hesitancy and foster vaccine confidence. For example, Chou and Budenz (2020) suggested that “attending to negative emotions such as fear and anxiety, raising awareness of emotional manipulations by anti-vaccine disinformation efforts, and activating positive emotions such as altruism and hope” (p. 1718) might lower the reluctance to getting COVID-19 vaccinated. In the current research, higher levels of TP were significantly associated with CBs. However, TP generally implies lower levels of control, and as Whitson and Galinsky (2008) suggested, lacking control increases illusory pattern perception, i.e., CBs. Generally, control deprivation and lack of certainty generate the attempt of people to restore it, and one possible way to do that is by seeking sources of external control (e.g., CB endorsement) (Kay et al., 2009; Landau et al., 2015). Therefore, we considered that effective strategies aimed to decrease vaccination hesitancy should also focus on increasing the sense of control of people concerning the SARS-CoV-2 by pointing out the role of vaccines in this regard.

This study has several limitations that need to be addressed. Among others, we considered that one of the most important limitations is the low sample of participants, which lowers the generalizability of this research. According to Crossman (2018) and Etikan (2016), convenient samples, such as the one we used, lower the ability to control the representativeness of the research group. Thus, for a more comprehensive view on vaccination acceptance/hesitancy among Romanian adults, further studies are needed, with larger and more heterogeneous samples, and if possible, using more specific survey methods. Our sample was formed almost exclusively by women, with a low percentage of men who answered the questions of our survey. Thus, the current gender-unbalanced sample also limits the generalizability of our findings. Furthermore, we considered that it is also important to acknowledge that the participants in our sample were adults from two cities from the northeastern side of Romania. Although there are no significant known regional variations in social, cultural, and educational attributes, this might also be considered in further studies on similar research topics.

Another limitation is related to the fact that we did not measure other personal variables, such as emotional states, personal experiences of COVID-19 (e.g., Did the participants get infected with the virus?), employment, income, trust in scientific experts, perceived vaccine safety, and source information of COVID-19 (e.g., the use of traditional and authoritative sources), as previous research confirmed their importance in studying attitudes and beliefs surrounding the novel coronavirus (Sherman et al., 2020; Guidry et al., 2021; Karlsson et al., 2021; Murphy et al., 2021; Thaker, 2021). Finally, we used a cross-sectional design, which did not allow us to make causal considerations. Future studies might consider longitudinal and more in-depth experimental approaches in order to establish potential associations between the changes in attitudes and beliefs of people concerning SARS-CoV-2 vaccination and worldwide vaccination outcomes.

## Conclusion

We considered that the current exploratory study, despite its limitations, contributes to a more comprehensive understanding of the psychological mechanisms related to vaccine hesitancy among Romanian adults. The current findings might be a valuable starting point in understanding the psychological constructs related to the extended TPB model and other personal factors and addressing the attitudinal roots that shape the acceptance and rejection of COVID-19 vaccination. Doing so would help tailor efficient public health messages through appropriate psychologically oriented approaches that would eventually increase COVID-19 vaccination awareness, understanding, and adherence.

## Data Availability Statement

The raw data supporting the conclusions of this article will be made available by the authors, without undue reservation.

## Ethics Statement

The studies involving human participants were reviewed and approved by Department of Psychology, Faculty of Psychology and Education Sciences, Alexandru Ioan Cuza University of Iaşi. The patients/participants provided their written informed consent to participate in this study.

## Author Contributions

Both authors conceived and designed the primary goal of the study, analyzed the data, and wrote the manuscript.

## Conflict of Interest

The authors declare that the research was conducted in the absence of any commercial or financial relationships that could be construed as a potential conflict of interest.

## Publisher's Note

All claims expressed in this article are solely those of the authors and do not necessarily represent those of their affiliated organizations, or those of the publisher, the editors and the reviewers. Any product that may be evaluated in this article, or claim that may be made by its manufacturer, is not guaranteed or endorsed by the publisher.

## References

[B1] Adiyoso W. Wilopo W. (2020). Social Distancing Intentions to Reduce the Spread of COVID19: The Extended Theory of Planned Behavior. Research Square. Available online at: https://assets.researchsquare.com/files/rs-61524/v1/80359e09-197c-400e-968b-ea736bd250a4.pdf 10.1186/s12889-021-11884-5 PMC8503732 34635071

[B2] Ajzen I. (2011). The theory of planned behaviour: reactions and reflections. Psychol. Health 26, 1113–1127. 10.1080/08870446.2011.613995 21929476

[B3] Allington D. Duffy B. Wessely S. Dhavan N. Rubin J. (2020). Health-protective behaviour, social media usag and conspiracy belief during the COVID-19 public health emergency. Psychol. Med. 10.1017/S003329172000224X. [Epub ahead of print]. 32513320 PMC7298098

[B4] Al-Mohaithef M. Padhi B. K. (2020). Determinants of COVID-19 vaccine acceptance in Saudi Arabia: a web-based national survey. J. Multidiscip. Healthc. 13, 1657–1663. 10.2147/JMDH.S276771 33262600 PMC7686470

[B5] Alper S. Bayrak F. Yilmaz O. (2020). All the Dark Triad and Some of the Big Five Traits are Visible in the Face. 10.31234/osf.io/c3ngz

[B6] Arafat Y. Izham M. Ibrahim M. (2018). The use of measurements and health behavioral models to improve medication adherence,” in Social and Administrative Aspects of Pharmacy in Low-and Middle-Income Countries, eds Izham M. Ibrahim M. Wertheimer A. I. Babar Z.-Ud.-D. (Doha: Academic Press), 53–69. 10.1016/B978-0-12-811228-1.00004-2

[B7] Barua Z. Barua S. Aktar S. Kabir N. Li M. (2020). Effects of misinformation on COVID-19 individual responses and recommendations for resilience of disastrous consequences of misinformation. Progress Disaster Sci. 8:100119. 10.1016/j.pdisas.2020.100119 34173443 PMC7373041

[B8] Bertin P. Nera K. Delouvée S. (2020). Conspiracy beliefs, rejection of vaccination, and support for hydroxychloroquine: a conceptual replication-extension in the COVID-19 pandemic context. Front. Psychol. 11:565128. 10.3389/fpsyg.2020.565128 33071892 PMC7536556

[B9] Biddlestone M. Green R. Douglas K. M. (2020). Cultural orientation, power, belief in conspiracy theories, and intentions to reduce the spread of COVID-19. Br. J. Soc. Psychol. 59, 663–673. 10.1111/bjso.12397 32592420 PMC7361833

[B10] Bierwiaczonek K. Kunst J. R. Pich O. (2020). Belief in COVID-19 conspiracy theories reduces social distancing over time. Appl. Psychol. Health Well Being 12, 1270–1285. 10.1111/aphw.12223 32864837

[B11] Bloomberg (2020). More Than 98.3 Million Shots Given: Covid-19 Tracker. Available online at: https://www.bloomberg.com/graphics/covid-vaccine-tracker-global-distribution/?sref=aSmaY14p&s=07&fbclid=IwAR0Voj9V8K_XRSn8RCQu5FFzPxK5~BYr5BNqNcC7PW5dDYD_PddJg6MtOumg

[B12] Brotherton R. French C. C. Pickering A. D. (2013). Measuring belief in conspiracy t heories: the generic conspiracist beliefs scale. Front. Psychol. 4:279. 10.3389/fpsyg.2013.00279 23734136 PMC3659314

[B13] Chou W. S. Budenz A. (2020). Considering emotion in COVID-19 vaccine communication: addressing vaccine hesitancy and fostering vaccine confidence. Health Commun. 35, 1718–1722. 10.1080/10410236.2020.1838096 33124475

[B14] Crossman A. (2018). Convenience Samples for Research. Thought Co. Available online at: http://www.thoughtco.com/convenience-sampling-3026726

[B15] Douglas K. M. Uscinski J. E. Sutton R. M. Cichocka A. Nefes T. Ang C. S.. (2019). Understanding conspiracy theories. Polit. Psychol. 40, 3–35. 10.1111/pops.12568

[B16] Earnshaw V. A. Eaton L. A. Kalichman S. C. Brousseau N. M. Hill E. C. Fox A. B. (2020). COVID-19 conspiracy beliefs, health behaviors, and policy support. Transl. Behav. Med. 10, 850–856. 10.1093/tbm/ibaa090 32910819 PMC7499784

[B17] Etikan I. (2016). Comparison of convenience sampling and purposive sampling. Am. J. Theor. Appl. Stat. 5, 1–10. 10.11648/j.ajtas.20160501.1124899564

[B18] Faul F. Erdfelder E. Lang A.-G. Buchner A. (2007). G^*^ Power 3: a flexible statistical power analysis program for the social, behavioral, and biomedical sciences. Behav. Res. Methods 39, 175–191. 10.3758/BF03193146 17695343

[B19] Fisher K. A. Bloomstone S. J. Walder J. Crawford S. Fouayzi H. Mazor K. M. (2020). Attitudes toward a potential SARS-CoV-2 vaccine: a survey of US adults. Ann. Intern. Med. 173, 964–973. 10.7326/M20-3569 32886525 PMC7505019

[B20] Floyd D. L. Prentice-Dunn S. Rogers R. W. (2000). A meta-analysis of research on protection motivation theory. J. Appl. Soc. Psychol. 30:407–429. 10.1111/j.1559-1816.2000.tb02323.x

[B21] Freeman D. Waite F. Rosebrock L. Petit A. Causier C. East A.. (2020). Coronavirus conspiracy beliefs, mistrust, and compliance with government guidelines in England. Psychol. Med. 10.1017/S0033291720001890. [Epub ahead of print]. 32436485 PMC7264452

[B22] Fu C. Wei Z. Pei S. Li S. Sun X. Liu P. (2020). Acceptance and preference for COVID- 19 vaccination in health-care workers (HCWs). medRxiv. 10.1101/2020.04.09.20060103

[B23] Gidengil C. A. Parker A. M. Zikmund-Fisher B. J. (2012). Trends in risk perceptions and vaccination intentions: a longitudinal study of the first year of the H1N1 pandemic. Am. J. Public Health 102, 672–679. 10.2105/AJPH.2011.300407 22397349 PMC3297965

[B24] GSSC Avangarde (2020). National Barometer. Available online at: https://drive.google.com/file/d/1WMXOORGCzVDQjzzp3oshYY24RVeC8HRe/view

[B25] Guidry J. Laestadius L. I. Vraga E. K. Miller C. A. Perrin P. B. Burton C. W.. (2021). Willingness to get the COVID-19 vaccine with and without emergency use authorization. Am. J. Infect. Control 49, 137–142. 10.1016/j.ajic.2020.11.018 33227323 PMC7677682

[B26] Hayes A. F. (2013). Introduction to Mediation, Moderation, and Conditional Process Analysis: A Regression-Based Approach. New York, NY: Guilford Press.

[B27] Imhoff R. Lamberty P. (2020). A bioweapon or a hoax? The link between distinct conspiracy beliefs about the coronavirus disease (COVID-19) outbreak and pandemic behavior. Soc. Psychol. Personal. Sci. 11, 1110–1118. 10.1177/1948550620934692 PMC7342934 38602949

[B28] IPSOS (2020). Romanians, on the Last Places in the World Regarding the Intention to Vaccinate Against COVID-19, If the Vaccine Were Available. Available online at: https://www.ipsos.com/ro-ro/romanii-pe-ultimele-locuri-lume-ceea-ce-priveste-intentia-de-vaccinare-anti-covid-19-daca-vaccinul

[B29] Jolley D. Douglas K. M. (2014). The effects of anti-vaccine conspiracy theories on vaccination intentions. PLoS ONE 9:e89177. 10.1371/journal.pone.0089177 24586574 PMC3930676

[B30] Karlsson L. C. Soveri A. Lewandowsky S. Karlsson L. Karlsson H. Nolvi S.. (2021). Fearing the disease or the vaccine: the case of COVID-19. Pers. Individ. Dif. 172:110590. 10.1016/j.paid.2020.110590 33518869 PMC7832025

[B31] Kay A. C. Whitson J. A. Gaucher D. Galinsky A. D. (2009). Compensatory control: achieving order through the mind, our institutions, and the heavens. Curr. Dir. Psychol. Sci. 18:264–268. 10.1111/j.1467-8721.2009.01649.x

[B32] Kim S. Kim S. (2020). Searching for general model of conspiracy theories and its implication for public health policy: analysis of the impacts of political, psychological, structural factors on conspiracy beliefs about the COVID-19 pandemic. Int. J. Environ. Res. Public Health 18:266. 10.3390/ijerph18010266 33396494 PMC7796329

[B33] Landau M. J. Kay A. C. Whitson J. A. (2015). Compensatory control and the appeal of a structured world. Psychol. Bull. 141, 694–722. 10.1037/a0038703 25688696

[B34] Lewandowsky S. Gignac G. E. Oberauer K. (2013). The role of conspiracist ideation and worldviews in predicting rejection of science. PLoS ONE 8:e75637. 10.1371/journal.pone.0075637 24098391 PMC3788812

[B35] Lin C. Tu P. Beitsch L. M. (2020). Confidence and receptivity for COVID-19 vaccines: a rapid systematic review. Vaccines 9:16. 10.3390/vaccines9010016 33396832 PMC7823859

[B36] Maftei A. Holman A. C. (2020). Beliefs in conspiracy theories, intolerance of uncertainty, and moral disengagement during the coronavirus crisis. Ethics Behav. 10.1080/10508422.2020.1843171

[B37] Murphy J. Vallières F. Bentall R. P. Shevlin M. McBride O. Hartman T. K.. (2021). Psychological characteristics associated with COVID-19 vaccine hesitancy and resistance in Ireland and the United Kingdom. Nat. Commun. 12:29. 10.1038/s41467-020-20226-9 33397962 PMC7782692

[B38] Neumann-Böhme S. Varghese N. E. Sabat I. Barros P. P. Brouwer W. van Exel J.. (2020). Once we have it, will we use it? A European survey on willingness to be vaccinated against COVID-19. Euro. J. Health Econ. Health Econ. Prev. Care 21, 977–982. 10.1007/s10198-020-01208-6 32591957 PMC7317261

[B39] Nguyen T. Henningsen K. H. Brehaut J. C. Hoe E. Wilson K. (2011). Acceptance of a pandemic influenza vaccine: a systematic review of surveys of the general public. Infect. Drug Resist. 4, 197–207. 10.2147/IDR.S23174 22114512 PMC3215344

[B40] Orosz G. Krekó P. Paskuj B. Tóth-Király I. Bothe B. Roland-Lévy C. (2016). Changing Conspiracy Beliefs through Rationality and Ridiculing. Front. Psychol. 7, 1525. 10.3389/fpsyg.2016.01525 27790164 PMC5061726

[B41] Pan Y. Fang Y. Xin M. Dong W. Zhou L. Hou Q.. (2020). Self-reported compliance with personal preventive measures among chinese factory workers at the beginning of work resumption following the COVID-19 outbreak: cross-sectional survey study. J. Med. Internet Res. 22:e22457. 10.2196/22457 32924947 PMC7527164

[B42] Pascual-Leone A. Cattaneo G. Macià D. Solana J. Tormos J. M. Bartrés-Faz D. (2021). Beware of optimism bias in the context of the COVID-19 pandemic. Ann. Neurol. 89, 423–425. 10.1002/ana.26001 33426696 PMC8014543

[B43] Paul E. Steptoe A. Fancourt D. (2020). Attitudes towards vaccines and intention to vaccinate against COVID-19: Implications for public health communications. Lancet Regional Health Europe. 10.1016/j.lanepe.2020.100012. [Epub ahead of print]. 33954296 PMC7834475

[B44] Romer D. Jamieson K. H. (2020). Conspiracy theories as barriers to controlling the spread of COVID-19 in the US. Soc. Sci. Med. 263:113356. 10.1016/j.socscimed.2020.113356 32967786 PMC7502362

[B45] Sallam M. Dababseh D. Eid H. Al-Mahzoum K. Al-Haidar A. Taim D.. (2021). High rates of COVID-19 vaccine hesitancy and its association with conspiracy beliefs: a study in Jordan and Kuwait among other Arab countries. Vaccines 9:42. 10.3390/vaccines9010042 33445581 PMC7826844

[B46] Sherman S. M. Smith L. E. Sim J. Amlôt R. Cutts M. Dasch H.. (2020). COVID-19 vaccination intention in the UK: results from the COVID-19 vaccination acceptability study (CoVAccS), a nationally representative cross-sectional survey. Hum. Vaccines Immunother. 17, 1612–1621. 10.1080/21645515.2020.1846397 33242386 PMC8115754

[B47] Strecher V. J. Rosenstock I. M. (1997). The health belief model, in Health Behavior and Health Education: Theory, Research and Practice, eds Glanz K. Lewis F. M. Rimer B. (San Francisco, CA: Jossey-Bass), 41–59.

[B48] Taha S. A. Matheson K. Anisman H. (2013). The 2009 H1N1 influenza pandemic: the role of threat, coping, and media trust on vaccination intentions in Canada. J. Health Commun. 18, 278–290. 10.1080/10810730.2012.727960 23301849

[B49] Taylor S. Landry C. A. Paluszek M. M. Groenewoud R. Rachor G. S. Asmundson G. (2020). A proactive approach for managing COVID-19: the importance of understanding the motivational roots of vaccination hesitancy for SARS-CoV2. Front. Psychol. 11:575950. 10.3389/fpsyg.2020.575950 33192883 PMC7604422

[B50] Teovanović P. Lukić P. Zupan Z. Lazić A. Ninković M. ŽeŽelj I. (2020). Irrational beliefs differentially predict adherence to guidelines and pseudoscientific practices during the COVID-19 pandemic. Appl. Cogn. Psychol. 35, 486–496. 10.31234/osf.io/gefhn 33362344 PMC7753549

[B51] Thaker J. (2021). The persistence of vaccine hesitancy: COVID-19 vaccination intention in 498 New Zealand. J. Health Commun. 26, 104–111. 10.1101/2020.12.16.20248139 33719898

[B52] Whitson J. Galinsky A. (2008). Lacking control increases illusory pattern perception. Science 322, 115–117. 10.1126/science.1159845 18832647

[B53] World Health Organization (2019). Ten Threats to Global Health in 2019. Available online at: https://www.who.int/emergencies/ten-threats-to-global-health-in-2019 (accessed February 01, 2021).

[B54] Xu H. Gan Y. Zheng D. Wu B. Zhu X.. (2020). Relationship between COVID-19 infection and risk perception, knowledge, attitude, and four nonpharmaceutical interventions during the late period of the COVID-19 epidemic in china: online cross-sectional survey of 8158 adults. J. Med. Internet Res. 22:e21372. 10.2196/21372 33108317 PMC7669364

